# Effectiveness and safety of treatments for new-onset, refractory and relapsed vitreoretinal lymphoma: protocol for a systematic review and network meta-analysis

**DOI:** 10.1136/bmjopen-2025-115420

**Published:** 2026-10-08

**Authors:** Melissa K Shields, Richard J Woodman, Stewart R Lake, Laura E Downie, Justine R Smith

**Affiliations:** 1 Flinders Health and Medical Research Institute, and College of Medicine and Public Health, Flinders University, Adelaide, South Australia, Australia; 2 Department of Optometry and Vision Sciences, The University of Melbourne, Parkvile, Victoria, Australia

**Keywords:** Lymphoma, OPHTHALMOLOGY, Disease Management

## Abstract

**Abstract:**

**Introduction:**

Vitreoretinal lymphoma (VRL) is a rare intraocular malignancy. It has a poor prognosis because, despite a range of therapeutic approaches, central nervous system (CNS) lymphoma may arise at any stage. Because of the rarity of VRL, intervention studies that attempt to define optimal treatment, especially for refractory or relapsed disease, are small and dominated by retrospective and observational designs. Many treatment regimens are associated with substantial toxicity. A systematic review protocol has been developed to examine the existing literature on the effectiveness and safety of VRL-directed therapies in patients with newly diagnosed, refractory and relapsed cancer.

**Methods and analysis:**

Comprehensive literature searches will identify studies reporting on the treatment of VRL from four electronic bibliographic databases (Ovid MEDLINE, Ovid Embase, Scopus and Cochrane Central Register of Controlled Trials), in addition to the World Health Organization International Clinical Trials Registry Platform and ClinicalTrials.gov, from the year 2000. Reference lists of included studies will also be screened. There will be no language restrictions. Studies that enrolled men and women of any age with VRL (new-onset, refractory and relapsed), including both primary VRL and VRL with past or concurrent CNS involvement, will be included. The systematic review will encompass all VRL-directed ocular and systemic interventions evaluated in randomised controlled trials and observational studies. Studies that evaluated treatment of CNS lymphoma without ocular involvement, ocular cancers other than VRL, and inflammatory ocular diseases, studies that used topical medications only and experimental studies that employed animal models will be excluded. Two authors will independently screen titles and abstracts, followed by full-text eligibility assessment. Included studies will undergo data extraction and independent risk-of-bias assessment, with certainty of evidence appraised using the Grading of Recommendations Assessment, Development and Evaluation approach. A network meta-analysis will compare interventions directly or indirectly, as the data allow.

**Ethics and dissemination:**

Ethical approval is not required for the review as it relies on previously conducted studies. The findings will be disseminated in a peer-reviewed journal.

**PROSPERO registration number:**

CRD420251251269

**STRENGTHS AND LIMITATIONS OF THIS STUDY**

- A systematic review applying a network meta-analysis to treatments of new-onset, refractory and relapsed vitreoretinal lymphoma, with registration of the review protocol in PROSPERO.
- Comprehensive searches, conducted across four electronic bibliographic databases and two clinical trial registries.
- Independent double-screening and adjudication of citations to minimise selection bias.
- Numbers of studies of new immunotherapeutic and targeted drugs may be small.
- Inclusion of retrospective observational studies and single-centre study designs may introduce bias and limit generalisability.

## Introduction

 Vitreoretinal lymphoma (VRL) is an aggressive extranodal non-Hodgkin’s lymphoma characterised by infiltration of the vitreous, retina and/or optic nerve.^1^ This subset of primary central nervous system lymphoma (PCNSL) presents a substantial management challenge despite advances in diagnostics and treatment options with novel targets.^2 3^ VRL exhibits a variable pattern of central nervous system (CNS) involvement that may precede, coincide with or follow the ocular disease, and ultimately occurs in an estimated 69% of patients.^4^ Conversely, VRL occurs in about one in six patients with PCNSL.^4^ When VRL is confined to the eye at diagnosis, termed primary VRL (PVRL), around 50% of individuals experience progression of the cancer into the CNS.^5^ Overall survival remains poor, with the 5-year survival measured at approximately 40% in population-based studies.^6 7^ Existing data from treatment studies are methodologically heterogenous, making it difficult to ascertain the approach that achieves best outcomes.

Although VRL is the most common intraocular lymphoma, it is a rare cancer, accounting for less than 3% of all ocular tumours.^8^ The 10-year cumulative prevalence of PVRL was estimated at 0.076 per 100 000 persons using data from the TriNetX clinical database.^9^ VRL frequently masquerades as inflammatory eye disease, leading to diagnostic delays and misdiagnosis that further contribute to a poor prognosis.^1 2 5^ Treatment delays are associated with higher disease burden, including retinal and subretinal infiltration, which are significant predictors of vision loss^10 11^ and may be associated with shorter mean survival time.^12^ The optimal management of VRL continues to be debated, with the rarity of the disease making it difficult to recruit large cohorts for prospective clinical trials.

For patients with VRL, particularly those with relapsed or refractory disease, there is no standard treatment, and management decisions are determined by patient-specific factors, including age and comorbidities including frailty, as well as local expertise and resources.^2^ Management of VRL aims to eradicate the intraocular tumour, prevent progression to CNS involvement, reduce the relapse rate and improve overall survival.^3 13^ In a report from the International Vitreoretinal B-cell Lymphoma Registry, approximately one-half of the 80 patients with VRL received ocular treatment alone, while another quarter underwent combined ocular and extraocular treatment.^14^

Ocular treatments include chemotherapy and external beam radiotherapy. Ocular chemotherapy involves repeated intravitreal injections of a lymphotoxic drug, most commonly methotrexate, an antimetabolite, but also other agents, such as rituximab, an anti-CD20 monoclonal antibody.^15^ These local treatments can achieve high initial complete-response rates, and there may be an improvement in visual acuity.^16^ However, relapse of the VRL may occur.^17 18^ Adverse events depend on the drug: intravitreal methotrexate injections may cause corneal epitheliopathy, angle neovascularisation associated with elevated intraocular pressure and sterile endophthalmitis.^16^ Some centres combine intravitreal methotrexate with intravenous high-dose methotrexate and report efficacy and acceptable toxicity of the approach.^19^ Although it is a less popular local treatment,^14^ external beam ocular radiotherapy is reported to have good initial complete response rates.^20 21^ There is potential for ocular morbidity, including radiation retinopathy and cataract, more commonly at higher radiation doses,^22^ and relapses and CNS progression are still observed. Ocular radiotherapy also has been combined with methotrexate-based systemic chemotherapy with the goal of reducing CNS progression and prolonging survival.^23^

Systemic chemotherapy regimens usually include multiple drugs, often based around high-dose methotrexate.^19 24 25^ Induction, consolidation and maintenance protocols vary, and ocular treatments may be coadministered. The role of systemic chemotherapy in preventing or delaying CNS progression and improving survival remains uncertain. A systematic review and meta-analysis published in 2025 identified an association between systemic treatment and progression-free and overall survival in patients with VRL.^26^ Another systematic review and meta-analysis, published in the same year, indicated that in patients with PVRL, ocular treatment alone reduced the risk of developing CNS lymphoma compared with systemic or combined ocular and systemic treatment, with no difference in overall survival between groups.^27^ Systemic treatments can have serious toxicities, which may even be fatal, and this is often a consideration for patients with comorbidities. Additional approaches that are used for consolidation after systemic chemotherapy and/or as adjunctive treatments include autologous stem cell transplantation (ASCT) in younger patients, whole-brain radiation therapy (WBRT) and intrathecal chemotherapy.^28 28 29^

For patients experiencing relapsed VRL or whose disease is resistant to traditional regimens, treatment often centres on intensive systemic chemotherapy and ASCT, although with high rates of CNS progression.^30 31^ Intravitreal rituximab has been used as salvage therapy in a cohort previously treated with external beam ocular radiotherapy plus systemic treatments.^32^ Emerging biologic and targeted treatments include Bruton tyrosine kinase (BTK) inhibitors and a range of immunomodulatory drugs and immunotherapeutics, such as lenalidomide, checkpoint inhibitors and anti-CD19 chimeric antigen receptor (CAR) T-cell therapies.^33–38^ Among these, the BTK inhibitor, ibrutinib, has demonstrated clinical activity in patients with PCNSL in all CNS compartments, including the eyes as well as the brain parenchyma and cerebrospinal fluid, and long-lasting complete responses have been reported.^39 40^ Although promising and generally well tolerated, BTK inhibitors require broader validation, as studies have so far been limited by small sample sizes and short follow-up.

Previous systematic reviews and meta-analyses have sought to define the optimal treatment for VRL. These works have been narrowly scoped, including only PVRL^27^ or only first-line treatments,^41^ or focused primarily on effectiveness outcomes.^26 27^ Broad treatment-agnostic categories (eg, ocular-only vs ocular plus systemic) are frequently applied.^27 42^ A network meta-analysis (NMA) approach has not been applied to this topic previously, but could help move beyond such groupings.

This systematic review will evaluate the effectiveness and safety of local and systemic treatments for VRL across new-onset, refractory and relapsed presentations. With multiple therapeutic options and no certainty of the optimal approach, management remains highly challenging, especially for refractory and relapsed cases. Given the differences in conclusions around treatment effectiveness between reports and the evolving use of oral targeted drugs, a new systematic review, including an NMA, is indicated. The findings are expected to support clinical-decision making, inform new clinical guidelines and highlight gaps that define priorities for future research in the field.

### Review question

The question to be addressed in the planned systematic review will be: in patients of any age with newly diagnosed, refractory and relapsed VRL, what are the effectiveness and safety of all local and systemic VRL-directed interventions? Interventions are inclusive of intravitreal chemotherapy or immunotherapy, ocular radiotherapy, systemic chemotherapy, WBRT, ASCT and systemic immunotherapies and targeted drugs.

## Methods and analysis

The protocol has been registered with the PROSPERO International Prospective Register of Systematic Reviews (CRD420251251269). Major protocol amendments will be outlined in the systematic and NMA publication. The methodological approach is outlined using the Population, Intervention, Comparator and Outcomes framework in table 1.

**Table 1:** Population, Intervention, Comparator and Outcomes (PICO) framework applied to the systematic review and network meta-analysis of effectiveness and safety of treatments for new-onset, refractory and relapsed vitreoretinal lymphoma (VRL)

PICO tool	Description
Population	Patients of any age and sex or gender who are diagnosed with VRL that is newly diagnosed (treatment-naïve), refractory and relapsed, including VRL confined to the eye at diagnosis and VRL with past or concurrent CNS lymphoma.
Intervention	All VRL-directed ocular and systemic treatments excluding glucocorticoids and topically applied drugs.
Comparison	VRL-directed interventions will be compared in an NMA. When available, controls including observational or best-supportive care will be compared against VRL-directed interventions.
Outcome	Primary outcomes: median overall survival, median progression-free survival. Secondary outcomes: median disease-free survival, complete ocular response rate, partial ocular response rate, CTCAE Grade 3–4 adverse events.

CNS, central nervous system; CTCAE, Common Terminology Criteria for Adverse Events; NMA, network meta-analysis.

### Inclusion criteria

#### Study populations

The population will include men and women, or male and female, patients with new-onset (treatment-naïve), refractory and relapsed VRL, including PVRL or VRL with past or concurrent CNS involvement. Patients with relapsed VRL are defined as those who have previously received VRL-directed therapy and subsequently experienced local, CNS or systemic relapse following complete or partial response. Patients with refractory VRL are defined as those who did not respond adequately to standard initial treatment (ie, no response or progressive disease). CNS relapse is inclusive of any in-field, marginal, local, and distant brain, spinal cord and meningeal relapses. There will be no age-based eligibility restrictions.

#### Interventions

The review will include all VRL-directed ocular and systemic interventions evaluated in clinical trials, irrespective of dosage or route, to allow cross-modal comparisons across first-line, refractory and relapsed VRL. Treatments will be classified by: (1) therapeutic class (see itemised lists for local and systemic therapies), (2) treatment intent (first-line, maintenance, relapsed and refractory), (3) treatment modality (monotherapy and multimodal) and (4) sequence of administration. Active comparators will be clearly delineated.

Ocular therapies will include intravitreal methotrexate, intravitreal rituximab, intravitreal melphalan, other intravitreal drugs and external beam ocular radiotherapy. Subgroup analysis will compare cohorts receiving local ocular therapies with and without concurrent systemic therapies.

Systemic therapies will include systemic chemotherapy, BTK inhibitors (eg, ibrutinib, zanubrutinib, orelabrutinib), checkpoint inhibitors (eg, durvalumab, nivolumab), other systemic treatment options (eg, lenalidomide, pomalidomide) with or without rituximab, CAR T-cell therapy, ASCT with or without other regimens, and WBRT with or without other regimens.

#### Comparators

Clinical trials that evaluate VRL-directed interventions against a defined comparator group (eg, placebo, no treatment and another VRL-targeted intervention) will be included. Studies with observation only (no treatment) and/or best supportive care comparative arms, and studies without comparators will also be included. No-treatment and best supportive care will be grouped together as a single comparator for the analysis.

#### Study designs

Clinical trials, both randomised and non-randomised, as well as observational studies, such as retrospective cohorts, cross-sectional studies and case series, will be included. Initially, all reports will be considered; however, if eligible studies include more than 50 patients, case reports and case series with less than 5 patients may be excluded. Eligible studies will encompass all publications from the year 2000. No restrictions will be applied based on the language or setting of the study. In cases of overlapping reports from the same clinical cohort, data will be extracted from the publication reporting the largest sample size. Published conference abstracts will also be considered eligible for inclusion.

### Exclusion criteria

Exclusion criteria will comprise studies of: (1) PCNSL without ocular involvement; (2) ocular comorbidities of inflammatory or neoplastic origin, other than VRL; (3) animal models or in vitro cell-based experiments; and (4) topical medications (ie, eye drops). Additionally, systematic reviews, meta-analyses, literature reviews, editorials and commentary articles will be excluded.

### Outcomes

The primary outcomes will be:

1. Median overall survival: median time from randomisation or initiation of primary therapy to death.^43^
2. Median progression-free survival: median time from randomisation or initiation of primary therapy to first evidence of disease progression or death, whichever comes first.^43^ Progressive ocular disease will be defined as an increase in the vitreous cells, or progressive retinal and/or optic nerve infiltration.^44^ Progressive CNS disease will be defined as a 25% or greater increase in the lesion volume on brain or spinal cord imaging compared with baseline or best response.^44^ The appearance of any new lesion(s) or disease site(s) during treatment also will qualify as progressive disease.

The secondary outcomes will be:

1. Median disease-free survival: time from randomisation or initiation of primary therapy to disease recurrence or relapse, where relapsed disease is defined as the appearance of any new lesion.^43 44^
2. Ocular complete response rate: no evidence of active VRL as defined by absence of cells in the vitreous and resolution of any previously documented retinal or optic nerve infiltrates.^44^
3. Ocular partial response rate: persistent, but decreased vitreous cells and retinal or optic nerve infiltrates.^44^
4. Common Terminology Criteria for Adverse Events (CTCAE) Grade 3–4 adverse event rates.^45^ Ocular and systemic events will be reported and analysed separately.
  - CTCAE Grade 3 adverse events. Ocular: best-corrected visual acuity worse than 6/12 or decreased more than three lines, or grade-defined ocular events. Severe or medically significant but not immediately life-threatening; hospitalisation or prolongation of hospitalisation indicated; disabling; limiting self-care activities of daily living.
  - CTCAE Grade 4 adverse events. Ocular: best-corrected visual acuity worse than 6/60. Systemic: life-threatening consequences; urgent intervention indicated.

If inconsistent terminology is used to define any outcome measure, outcomes will be classified according to the narrative descriptions that most closely match these definitions. Given the anticipated variability across studies in the definitions of key outcomes, author-reported definitions will be extracted and recorded alongside the corresponding data. If outcome definitions are not explicitly stated or diverge from those listed, this will be reported as a source of heterogeneity in the synthesis and interpretation of pooled findings.

Effect measures will consist of pooled proportions for single-arm studies and risk ratios (RRs) or odds ratios (ORs) with 95% confidence intervals (CIs) for comparative studies. Treatment effects will be assessed as change-from-baseline across prespecified follow-up periods for applicable outcomes. For each outcome, change-from-baseline estimates at early (<3 months), intermediate (3–6 months, >6–12 months), late (2 years (24 months) and 5 years (60 months)) time periods will be extracted; if outcomes at these prespecified timepoints are not provided, the nearest reported timepoint will be used. If the included studies consistently report alternative timepoints (eg, 12-month intervals), extraction will be harmonised to those timepoints for comparability. Data will be collected for adverse events during treatment and through to the last follow-up.

Minimally important difference thresholds have not been prespecified because no validated, disease-specific values exist for VRL given its rarity and clinical heterogeneity, and applying thresholds from unrelated contexts could misleadingly imply unsupported precision. Therefore, disease-specific thresholds will not be applied to determine clinical relevance. Instead, treatment effects will be interpreted in a pragmatic, expert-informed framework that incorporates the magnitude and precision of effect estimates, certainty of the evidence and the predicted balance of benefit and risk. Effects suggesting a meaningful improvement in overall survival or progression-free survival will be considered to have greater clinical importance, especially when little or no effect can be excluded by CIs, and the results are consistent across study designs or treatment subgroups. For ocular response outcomes, larger complete or partial response rates will be considered clinically relevant if responses are sustained and associated with reduced CNS progression or maintenance of visual acuity where documented. For safety outcomes, differences in CTCAE Grade 3–4 adverse events will be deemed clinically relevant if they represent a material increase in treatment burden, treatment stoppage and/or risk of life-threatening or sight-threatening toxicity. Where the data allow, these judgments will be applied individually for newly diagnosed, refractory and relapsed VRL groups.

For the NMA, treatments and head-to-head comparisons will be displayed as nodes and lines, respectively. Nodes will be sized to reflect participant numbers, with the thickness of the lines adjusted to represent the number of head-to-head comparisons. Direct comparisons will be shown on the lines. Pooled hazard ratios (HRs) (95% CI) will be reported for overall survival and progression-free survival, and pooled ORs (95% CI) will be reported for overall response rate and adverse events.

### Search strategy

Comprehensive database searches will be conducted in Ovid MEDLINE, Ovid Embase, Scopus and the Cochrane Central Register of Controlled Trials to identify articles published from the year 2000. The starting year boundary aligns with International Classification of Diseases code changes to ensure cases of VRL are identified consistently across time. In addition, trial registries including the World Health Organization International Clinical Trials Registry Platform and ClinicalTrials.gov will be interrogated. No language restrictions will be applied; non-English articles will be translated to English using Google Translate. The search strategy will incorporate VRL management and treatment outcome-related terms. Grey literature will not be sourced for this systematic review. Details of the proposed Ovid MEDLINE search can be found in the online supplementary material. Reference lists of all included studies will be screened to identify any potential additional relevant publications.

### Data management and collection

Citations retrieved from the searches will be transferred to the Zotero citation management system (Zotero Corporation for Digital Scholarship, Vienna, Virginia, USA) to perform automated deduplication and to identify any articles that have been subsequently retracted from publication. One reviewer will manually confirm Zotero-flagged duplicates to ensure accuracy. A master citation library of unique records will then be transferred to Covidence systematic review management software (Veritas Health Innovation, Melbourne, Australia) for study screening.

#### Screening

Two independent review authors will use Covidence to identify eligible studies. A two-stage process, consisting of simultaneous title and abstract screening, followed by full-text screening against the stated study eligibility criteria, will be performed. During the title and abstract screening phase, review authors will evaluate each study by voting ‘yes’, ‘no’ or ‘maybe’. Studies receiving an agreed ‘no’ vote will be eliminated from further consideration, while those with ‘yes’ and ‘maybe’ votes will advance to full-text review for comprehensive evaluation. Conflicts will be resolved, at both steps, via discussion between the two review authors. If consensus cannot be reached, an independent review author will adjudicate the eligibility determination. Reference mining of full text articles will be conducted, and potentially eligible studies will be assessed against the study eligibility criteria. Studies that require translation will be software tagged in Covidence. Articles that are not free-to-access will be retrieved via an institutional interlibrary loan service, and failing this, the authors will be contacted directly with a request to provide the article. Retrieval of articles as PDF files will be managed through Zotero. A pilot screening will be undertaken to ensure that the review authors understand the process and eligibility criteria. The same conflict resolution process will apply to the voting process for full-text articles as for title and abstract screening.

The flow of studies through the screening process, from searching through to full-text screening, will be presented in a Preferred Reporting Items for Systematic Reviews and Meta-Analyses (PRISMA) flow diagram.^46^ A hierarchical list of exclusion reasons will be established to minimise conflicts when multiple reasons for exclusion exist, with the order being: (1) intervention, (2) study population, (3) study design, (4) treatment dosing, if applicable and (5) setting. The PRISMA flow diagram will show the number of exclusions for each reason, which will be tracked in Covidence.

#### Data extraction and management

Data will be extracted independently by two review authors into table files in Excel, current version (Microsoft Corporation, Redmond, Washington, USA), with conflicts resolved by discussion and/or adjudication by a third reviewer if required. The following information will be sought from eligible studies:

1. Article details: first and corresponding author names; journal; year; article type (ie, randomised clinical trial, cohort study, case series); language; publication status; DOI.
2. Study details: clinical trial registration (if relevant); study dates; design (type, phase); number and location of centres; study name (if stated); whether authors were contacted for further data or clarification.
3. Methods: sample size; disease status (new-onset, relapsed, or refractory disease); extent of dissemination at diagnosis (localised to the eye or concurrent CNS disease), primary intervention; outcome measures; postrandomisation exclusions; median follow-up; loss to follow-up; handling of missing data.
4. Participants: number per intervention group; baseline characteristics; participant inclusion and exclusion criteria.
5. Interventions: intervention type and comparator(s); treatment duration; follow-up duration.
6. Treatment outcomes: prespecified primary and secondary outcomes.
7. Other: funding sources; conflicts of interest.

### Assessment of risk of bias in included studies and certainty of body of evidence

Risk of bias for each included study will be assessed with design-appropriate tools: Cochrane Risk of Bias 2^47^ for randomised trials; Cochrane Risk Of Bias In Non-randomized Studies - of Interventions^48^ for non-randomised clinical intervention studies; National Institutes of Health quality assessment tools^49^ for observational studies. Each study will be assessed for potential biases in areas such as selection, performance, detection, attrition, reporting and other sources of bias. Two review authors will independently assess the risk level in each domain, categorising it as low, high or unclear risk. Any disagreements will be resolved through discussion between the review authors and, if necessary, an independent adjudicator will be consulted to address any remaining discrepancies. For missing data, we will contact authors for clarification; if data remain unavailable at 4 weeks after initial contact, analyses will proceed using the available data from the full text publication. Risk-of-bias tables will be created in RevMan (The Cochrane Collaboration, London, UK).

The certainty of evidence will be assessed independently by two review authors using the Grading of Recommendations Assessment, Development and Evaluation (GRADE) approach, which classifies evidence certainty as high, moderate, low and very low, based on domains such as study design, risk of bias and inconsistency.^50^ Confidence In Network Meta-Analysis (or GRADE-NMA) will be used to rate the certainty of evidence for each pairwise comparison. This tool penalises reporting bias and imprecision, ensuring that highly ranked treatments with little data are flagged as having low or very low certainty.^51^ Summary of Findings tables will present GRADE judgements for each primary outcome.

Publication bias will be assessed using Risk Of Bias due to Missing Evidence in a Network meta-analysis^52^ in order to evaluate missing-evidence bias across the network and inform the certainty of the results. Additionally, network funnels (comparison-adjusted funnel plots) will be utilised to visually inspect the network for small-study effects and publication bias.

### Data synthesis and statistical analysis

#### Standard pairwise and single-proportion meta-analysis

The meta-analysis will be conducted using a hybrid approach to accommodate both single-arm and comparative multi-arm studies. Effect estimates will be synthesised separately for each outcome across studies, with treatments analysed within their pre-defined treatment categories. Studies will be grouped according to common treatment regimens (ie, intravitreal methotrexate in combination with intravenous high-dose methotrexate) to maintain clinical comparability.

When a study reports more than one eligible comparison, the primary effect will be designated in accordance with the prespecified primary outcomes to avoid within-study dependence. The primary analysis will include all studies regardless of GRADE score. A secondary analysis will exclude studies judged to have a high risk of bias. Analysis will be done on a per-patient basis. The meta-analysis will be conducted in Stata (19.1, StataCorp LLC, College Station, Texas, USA).

For dichotomous outcomes (eg, complete or partial response, adverse events), pooled absolute proportions will be used for single-arm studies, and RRs or ORs with 95% CIs will be used for direct comparative studies. Single-arm studies will be analysed separately using proportional meta-analysis. Time-to-event outcomes will use pooled HRs when possible. Random-effects models will be applied when analysing more than three studies per category; otherwise, fixed-effects models will be used.

Treatment categories will be included in the analysis only when at least two studies report the treatment at a comparable dose and regimen. If dose intensity or schedule vary substantially between grouped studies within the same treatment category, sensitivity analyses will be performed excluding or stratifying by these subgroups to assess whether pooled findings are robust to the heterogeneity. The anticipated minimum treatment categories with sufficient data are: intravitreal methotrexate monotherapy; intravenous high-dose methotrexate-based therapy; intravitreal methotrexate in combination with intravenous high-dose methotrexate; ocular external beam radiotherapy monotherapy; ocular external beam radiotherapy with intravenous high-dose methotrexate; BTK inhibitors. Any dose and regimen will be accepted if at least one dose was administered. Additional treatment categories may be added as data become available. If sufficient data exist, individual BTK inhibitors will be treated as separate categories. If data are insufficient for individual analyses, BTK inhibitors will be pooled and a class-level analysis will be conducted. In cases of sparse data, ocular treatments (ocular external beam radiotherapy and intravitreal drugs) will be aggregated into the category “Ocular therapy”; all systemic treatments will be aggregated into the category “Systemic therapy”; and combinations of ocular and systemic treatments will be grouped as a “Combined” composite treatment category. More granular splits will be tested as data permit.

#### Network meta-analysis

For the subset of prospective and retrospective studies that are comparative (containing two or more distinct treatment arms) and share common treatment nodes, a random-effects NMA will be performed. The NMA will leverage both direct and indirect evidence to compare the relative efficacy and safety of the treatments and generate a hierarchical ranking using Surface Under the Cumulative RAnking scores.

Treatments for new-onset, relapsed and refractory cases will be placed into separate networks. The NMA methodology offers several distinct advantages over traditional pairwise meta-analysis, particularly in the context of rare conditions such as VRL where head-to-head trials may be sparse. By synthesising data from a connected network of studies, NMA allows for the estimation of relative treatment effects between interventions that have never been directly compared in clinical trials (indirect comparison).^53^ Furthermore, NMA combines direct and indirect evidence to increase the precision of treatment effect estimates and provides a unified hierarchy of treatments, enabling the ranking of interventions based on their probability of being the most effective. Crucially, unlike naive indirect comparisons, NMA preserves the benefits of randomisation by comparing treatments against a common comparator within trials, thus maintaining the internal validity of the included studies.^53^ By integrating both direct and indirect evidence, the method establishes comparative networks even for treatments lacking head-to-head clinical trials.^54^

#### Heterogeneity and transitivity assessment

Heterogeneity in random effect distributions will be evaluated using Cochran’s Q test, I^2^ (percentage of variation due to true heterogeneity) and τ² (estimated between-study variance).^55^ Significant heterogeneity will be defined as I^2^ greater than 50% together with Cochran’s Q test p<0.05 (based on χ^2^ distribution), indicating meaningful variation between studies rather than within-subject variability.

For the NMA component, the assumption of transitivity will be evaluated clinically by reviewing baseline patient demographics, disease status (new-onset vs relapsed and refractory), and definitions of supportive care across trials to ensure they are sufficiently similar to allow valid indirect comparisons. Inconsistency between direct and indirect evidence within the loop will be assessed using the side-splitting method. If significant heterogeneity or inconsistency is detected, network meta-regression or stratified subgroup analyses will be performed with moderators such as study design (retrospective vs prospective), baseline characteristics, geographical region, follow-up duration and risk-of-bias indicators.

#### Subgroup analysis

If at least two studies per intervention category are identified, subgroup analyses will be conducted to explore factors that may influence outcomes. The following subgroup analyses are planned to explore potential effect modifiers across VRL treatments: (1) by treatment modality (ocular therapies alone, systemic therapies alone or combination approaches); (2) by CNS involvement (no CNS disease vs VRL with CNS involvement); (3) by type of ocular therapy (intravitreal methotrexate, intravitreal rituximab or external beam ocular radiotherapy); (4) for BTK inhibitors vs systemic chemotherapy; (5) by concomitant administration of immunotherapy, evaluated only when immunotherapy is given alongside systemic chemotherapy (systemic chemotherapy plus systemic immunotherapy vs systemic chemotherapy alone); (6) by era; and (7) by prior therapy status (treatment naïve vs previously treated).

#### Sensitivity analysis

If sufficient data are available, sensitivity analyses will be performed on outcome measures to evaluate the impact of excluding studies with a high risk of bias in any single risk of bias domain (eg, studies funded by industry). A list of studies that have been excluded will be provided in the systematic review and NMA publication.

#### Summary of findings

Summary of Findings tables will be presented, comparing interventions’ effectiveness, safety and evidence quality according to the *Cochrane Handbook for Systematic Reviews*.^56^ NMA diagrams will be generated to visualise treatment interconnections, while forest plots and league tables will be used to display NMA estimates based on direct and indirect evidence.

### Patient and public involvement

None.

## Ethics and dissemination

This review does not require ethical approval, as it will use data from previously published studies. The findings will be disseminated in a peer-reviewed journal publication.

## Discussion

VRL is a rare, aggressive intraocular malignancy that continues to have a poor survival prognosis.^2 3^ Ocular treatments often yield good initial responses, but relapses may occur and CNS-related mortality remains high. The heterogeneity in management strategies underscores the need for consensus to optimise treatment pathways for improved outcomes of VRL.

Research focused on the outcomes of treatments for VRL faces multiple challenges related to the rarity of the disease. among these challenges, era effects are one key limitation. Over time, advances in ocular imaging and molecular diagnostics may impact case identification and thus outcome assessments.^57^ Consequently, dated cohorts may be differently classified or representative compared with more recent groups, potentially biasing comparisons across time. Where feasible, we will conduct era-aware analyses (era-stratified analyses or sensitivity checks), report any differences in findings across time periods, and consider the limitations these may impose on generalisability.

The evidence base for treatment of VRL is characterised by a predominance of retrospective, observational and/or single-centre study designs, which may introduce bias and limit the generalisability of findings. Compounding this, treatment heterogeneity represents another major challenge, arising largely from the absence of standardised guidelines for treatment dosing, regimens and combination strategies. Treatment protocols may differ between and within countries,^29^ driven by factors that could include region-specific practices and drug availability, as well as individual clinician preferences, and this issue may complicate cross-study comparisons and synthesis of the evidence. To address heterogeneity in treatment studies, we will use a random-effects framework with heterogeneity assessment (I², τ²) and prespecified meta-regression and subgroup analyses. We will review outcome definitions and treatment regimens across studies to ensure cross-study comparability. Where feasible, we will avoid a single ‘primary treatment’ analysis. We have also predefined treatment nodes to distinguish ocular vs systemic therapies and accommodate common combinations (eg, intravitreal methotrexate plus intravenous methotrexate).

In recent years, several novel drugs have been used to treat VRL.^33–36 58 59^ These treatments are still being introduced into clinical practice, and relevant reports may not yet be published. Because of sparse data, the network estimates for these therapies may inflate confidence in their perceived rankings. To counter this, we will apply certainty-of-evidence assessments and use network funnels to assess small-study effects and potential bias. Variations in patient demographics, healthcare infrastructure and background supportive care are possible effect modifiers in VRL. Where the data permit, we will perform network meta-regression and stratified subgroup analyses using these modifiers, with geographical region as a proxy for resource settings and supportive care standards.

This systematic review and NMA will evaluate the effectiveness and safety of treatments for new-onset, relapsed and refractory VRL. The NMA should, in particular, provide an effectiveness and safety hierarchy that can serve as a decision-support tool and perhaps boost interest in the development of clinical guidelines that can be used across global resource settings.

## Supplementary material

10.1136/bmjopen-2025-115420 online supplemental file 1

## References

[1] Levasseur SD, Wittenberg LA, White VA (2013). Vitreoretinal lymphoma: a 20-year review of incidence, clinical and cytologic features, treatment, and outcomes. JAMA Ophthalmol.

[2] Soussain C, Malaise D, Cassoux N (2021). Primary vitreoretinal lymphoma: a diagnostic and management challenge. Blood.

[3] Sobolewska B, Chee S-P, Zaguia F (2021). Vitreoretinal Lymphoma. Cancers (Basel).

[4] Farrall AL, Smith JR (2020). Eye involvement in primary central nervous system lymphoma. Surv Ophthalmol.

[5] Zhao X, Cheng T, Meng L (2022). Clinical Features, Diagnosis, Management and Prognosis of Primary Intraocular Lymphoma. Front Oncol.

[6] Farrall AL, Smith JR (2023). Incidence and survival of ocular diffuse large B‐cell lymphomas. Acta Ophthalmol (Copenh).

[7] Ahmed AH, Foster CS, Shields CL (2017). Association of Disease Location and Treatment With Survival in Diffuse Large B-Cell Lymphoma of the Eye and Ocular Adnexal Region. JAMA Ophthalmol.

[8] Malsch RM, Tessem RB, Dalvin LA (2025). Insights from the First Five Years of the Prospective Ocular Tumor Study. Semin Ophthalmol.

[9] Zhang A, Teplitsky D, Berkenstock MK (2025). Incidence, Prevalence, and Ocular Comorbidities of Primary Vitreoretinal Lymphoma in the TriNetX Global Collaborative Network. J Curr Ophthalmol.

[10] Kim M, Suh H, Park YG (2023). Clinical features predictive of vision loss in patients with vitreoretinal lymphoma: a single tertiary center experience. Sci Rep.

[11] Menean M, Cicinelli MV, Rivolta MC (2024). The Silent Masquerade: Clinical and Imaging Features of Asymptomatic Vitreoretinal Lymphoma. Am J Ophthalmol.

[12] Dalvin LA, Lim L-AS, Ancona-Lezama D (2020). Clinical Features Predictive of Survival in Patients With Vitreoretinal Lymphoma: Analysis of 70 Patients at a Single Ocular Oncology Center. Asia Pac J Ophthalmol (Phila).

[13] Chan C-C, Rubenstein JL, Coupland SE (2011). Primary vitreoretinal lymphoma: a report from an International Primary Central Nervous System Lymphoma Collaborative Group symposium. Oncologist.

[14] International Vitreoretinal B-Cell Lymphoma Registry Investigator Group (2024). Presentation, Diagnostic Testing and Initial Treatment of Vitreoretinal Lymphoma. Ophthalmol Retina.

[15] Kvopka M, Lake SR, Smith JR (2020). Intraocular chemotherapy for vitreoretinal lymphoma: A review. Clinical Exper Ophthalmology.

[16] Habot-Wilner Z, Frenkel S, Pe’er J (2021). Efficacy and safety of intravitreal methotrexate for vitreo-retinal lymphoma - 20 years of experience. Br J Haematol.

[17] Larkin KL, Saboo US, Comer GM (2014). Use of intravitreal rituximab for treatment of vitreoretinal lymphoma. Br J Ophthalmol.

[18] Smith JR, Rosenbaum JT, Wilson DJ (2002). Role of intravitreal methotrexate in the management of primary central nervous system lymphoma with ocular involvement. Ophthalmology.

[19] Cheng C-L, Yeh P-T, Fang W-Q (2023). Long-term outcomes of combined intravitreal methotrexate and systemic high-dose methotrexate therapy in vitreoretinal lymphoma. Cancer Med.

[20] Berenbom A, Davila RM, Lin H-S (2007). Treatment outcomes for primary intraocular lymphoma: implications for external beam radiotherapy. Eye (Lond).

[21] Mikami R, Nakayama H, Goto H (2013). Preliminary results of radiotherapy for primary intraocular non-Hodgkin lymphoma. Leuk Lymphoma.

[22] Milgrom SA, Cheah CY, Pinnix CC (2016). Acute and late toxicity of bilateral orbital irradiation in the management of primary intraocular lymphoma. Leuk Lymphoma.

[23] de la Fuente MI, Alderuccio JP, Reis IM (2019). Bilateral radiation therapy followed by methotrexate-based chemotherapy for primary vitreoretinal lymphoma. Am J Hematol.

[24] Kaburaki T, Taoka K, Matsuda J (2017). Combined intravitreal methotrexate and immunochemotherapy followed by reduced-dose whole-brain radiotherapy for newly diagnosed B-cell primary intraocular lymphoma. Br J Haematol.

[25] Klimova A, Heissigerova J, Rihova E (2018). Combined treatment of primary vitreoretinal lymphomas significantly prolongs the time to first relapse. Br J Ophthalmol.

[26] Gao J, Lv L-W, Yang J (2025). Exploration of the Optimal Treatment Modality for Vitreoretinal Lymphoma: A PRISMA Compliant Meta-Analysis and Systematic Review. Cancer Med.

[27] Ostrovsky M, Sela TC, Habot‐Wilner Z (2025). Comparison of various treatment modalities in patients with primary vitreoretinal lymphoma: A systematic review and meta‐analysis. Clinical Exper Ophthalmology.

[28] Pulido JS, Johnston PB, Nowakowski GS (2018). The diagnosis and treatment of primary vitreoretinal lymphoma: a review. Int J Retin Vitr.

[29] Riemens A, Bromberg J, Touitou V (2015). Treatment strategies in primary vitreoretinal lymphoma: a 17-center European collaborative study. JAMA Ophthalmol.

[30] Soussain C, Choquet S, Fourme E (2012). Intensive chemotherapy with thiotepa, busulfan and cyclophosphamide and hematopoietic stem cell rescue in relapsed or refractory primary central nervous system lymphoma and intraocular lymphoma: a retrospective study of 79 cases. Haematologica.

[31] Mainguy A, Soussain C, Touitou V (2025). High-dose chemotherapy with autologous haematopoietic stem cell transplantation in patients with isolated vitreoretinal lymphoma: a LOC network study. Bone Marrow Transplant.

[32] Cristescu I-E, Pagnacco C, Pershad S (2026). Successful use of intravitreal rituximab as salvage therapy for relapsed vitreoretinal lymphoma. Indian J Ophthalmol.

[33] Zhang Y, Zhang X, Zou D (2021). Lenalidomide and Rituximab Regimen Combined With Intravitreal Methotrexate Followed by Lenalidomide Maintenance for Primary Vitreoretinal Lymphoma: A Prospective Phase II Study. Front Oncol.

[34] Pivert C, Toutée A, Parizot C (2025). Preliminary evidence of CAR T-cell therapy activity in vitreoretinal lymphomas: An LOC network study. Hemasphere.

[35] Guan W, Wang L, Peng X (2022). Targeting Bruton’s tyrosine kinase in vitreoretinal lymphoma: an open-label, prospective, single-center, phase 2 study. Exp Hematol Oncol.

[36] Wang L, Guan W, Peng X (2021). Targeting Bruton Tyrosine Kinase With Zanubrutinib for Treatment of Vitreoretinal Lymphoma: Report of 3 Cases. Front Oncol.

[37] Ghesquieres H, Chevrier M, Laadhari M (2019). Lenalidomide in combination with intravenous rituximab (REVRI) in relapsed/refractory primary CNS lymphoma or primary intraocular lymphoma: a multicenter prospective “proof of concept” phase II study of the French Oculo-Cerebral lymphoma (LOC) Network and the Lymphoma Study Association (LYSA)†. Ann Oncol.

[38] Wang S-Y, Zhou S-W, Gao J (2025). Primary vitreoretinal lymphoma: diagnosis, treatment, and prognosis-a review of current knowledge and future directions. Blood Sci.

[39] Soussain C, Choquet S, Blonski M (2019). Ibrutinib monotherapy for relapse or refractory primary CNS lymphoma and primary vitreoretinal lymphoma: Final analysis of the phase II “proof-of-concept” iLOC study by the Lymphoma study association (LYSA) and the French oculo-cerebral lymphoma (LOC) network. Eur J Cancer.

[40] Soussain C, Malaise D, Choquet S (2023). Long-lasting CRs after ibrutinib monotherapy for relapse or refractory primary CNS lymphoma (PCNSL) and primary vitreoretinal lymphoma (PVRL): Long-term results of the iLOC study by the Lymphoma Study Association (LYSA) and the French Oculo-Cerebral Lymphoma (LOC) Network (clinical trial number: NCT02542514). Eur J Cancer.

[41] Gao J, Peng X, Wang L (2023). Efficacy and safety of first-line combination therapy versus monotherapy for vitreoretinal lymphoma: a systematic review and meta-analysis. BMC Ophthalmol.

[42] Raval V, Binkley E, Aronow ME (2021). Primary central nervous system lymphoma - ocular variant: an interdisciplinary review on management. Surv Ophthalmol.

[43] Delgado A, Guddati AK (2021). Clinical endpoints in oncology - a primer. Am J Cancer Res.

[44] Abrey LE, Batchelor TT, Ferreri AJM (2005). Report of an International Workshop to Standardize Baseline Evaluation and Response Criteria for Primary CNS Lymphoma. JCO.

[45] National Cancer Institute Common Terminology Criteria for Adverse Events (CTCAE) Version 6.0. U.S. https://ctep.cancer.gov/protocoldevelopment/electronic_applications/ctc.htm.

[46] Page MJ, McKenzie JE, Bossuyt PM (2021). The PRISMA 2020 statement: an updated guideline for reporting systematic reviews. BMJ.

[47] Sterne JAC, Savović J, Page MJ (2019). RoB 2: a revised tool for assessing risk of bias in randomised trials. BMJ.

[48] Sterne JA, Hernán MA, Reeves BC (2016). ROBINS-I: a tool for assessing risk of bias in non-randomised studies of interventions. BMJ.

[49] National Heart, Lung, and Blood Institute Study quality assessment tools. https://www.nhlbi.nih.gov/health-topics/study-quality-assessment-tools.

[50] Guyatt G, Oxman AD, Akl EA (2011). Grade guidelines: 1. Introduction-Grade evidence profiles and summary of findings tables. J Clin Epidemiol.

[51] Nikolakopoulou A, Higgins JPT, Papakonstantinou T (2020). Cinema: An approach for assessing confidence in the results of a network meta-analysis. PLoS Med.

[52] Chiocchia V, Nikolakopoulou A, Higgins JPT (2021). ROB-Men: a tool to assess risk of bias due to missing evidence in network meta-analysis. BMC Med.

[53] Woodman RJ (2011). Quantifying nonsteroidal anti-inflammatory drug (NSAID) risk: can combining the evidence with the use of a network meta-analysis provide a solution?. Ther Adv Drug Saf.

[54] Rouse B, Chaimani A, Li T (2017). Network meta-analysis: an introduction for clinicians. Intern Emerg Med.

[55] West SL, Gartlehner G, Mansfield AJ (2010). Table 7, Summary of common statistical approaches to test for heterogeneity. https://www.ncbi.nlm.nih.gov/books/NBK53317/table/ch3.t2/.

[56] Higgins JPT, Thomas J, Chandler J (2024). Cochrane Handbook for Systematic Reviews of Interventions version 6.5.

[57] Huang RS, Mihalache A, Popovic MM (2024). Diagnostic methods for primary vitreoretinal lymphoma: A systematic review. Surv Ophthalmol.

[58] Chaudhry AL, Chévez-Barrios P, Strati P (2025). Zanubrutinib is active for the treatment of relapsed primary vitreoretinal lymphoma. Leuk Lymphoma.

[59] Li Y, Qiao R, Zhang X (2026). Orelabrutinib and rituximab regimen combined with intravitreal methotrexate for treatment of primary vitreoretinal lymphoma: a case report and literature review. Front Oncol.

