# Supplementary material for: Effectiveness and safety of treatments for new-onset, refractory and relapsed vitreoretinal lymphoma: protocol for a systematic review and network meta-analysis
Source: BMJ Open. 2026 Oct 8;16(10):e115420. doi: 10.1136/bmjopen-2025-115420 (PMC13656148; doi:10.1136/bmjopen-2025-115420)
Supplement: Supplementary file 1 [file bmjopen-16-10-s001.pdf]

MEDLINE Search Strategy

Set	Search Statement
1	exp Lymphoma, Non-Hodgkin/ or exp Intraocular lymphoma/ or Eye Neoplasms/ or exp Lymphoma/
2	(((vitreoretinal* or intraocular* or ocular* or ophthalmic*) adj3 (lymphoma* or lymphoma:)) or PIOL or VRL or PVRL or PCNSL-O or ((PCNSL or "primary central nervous system lymphoma") adj3 (ocular* or intraocular* or ophthalmic* or eye*)) or ((DLBCL or lymphoma or b-cell) adj5 (eye* or ocular or intraocular* or vitreous or vitreal or retinal* or "posterior segment" or chorioretin*))).tw,kf.
3	1 or 2
4	clinical study/ or exp clinical trial/ or controlled clinical trial/ or exp randomized controlled trial/ or observational study/ or comparative study/ or multicenter study/
5	control groups/ or cross-over studies/ or double-blind method/ or matched-pair analysis/ or random allocation/ or single-blind method/ or Control Groups/ or Matched-Pair Analysis/ or prospective studies/ or retrospective studies/
6	clinical studies as topic/ or clinical trials as topic/ or case-control studies/ or retrospective studies/ or cohort studies/ or follow-up studies/ or prospective studies/ or controlled before-after studies/ or cross-sectional studies/ or interrupted time series analysis/ or multicenter studies as topic/ or case reports/
7	("clinical trial" or "clinical trial, phase i" or "clinical trial, phase ii" or clinical trial, phase iii or clinical trial, phase iv or controlled clinical trial or "multicenter study" or "randomized controlled trial" or clinical study or comparative study or controlled clinical trial or equivalence trial or observational study).pt.
8	(randomised or randomized or randomly or random or placebo or mask* or blind* or trial or placebo or quantitative or cohort or "4 arm" or "four arm" or baseline or consecutive* or "case series" or RCT).tw.
9	((quasi-experimental or clinical or cluster* or "before and after" or "before-and-after" or crossover or "cross-over" or "cross over" or retrospective or cohort or follow-up or "follow-up" or prospective or observation* or pre-post*) adj5 (trial* or studies or study)).tw.
10	(time* adj2 interrupted).tw.
11	(control* adj4 (Cohort* or group*)).tw.
12	(mixed-method* or mixed method* or (case* adj2 (series or report*))).tw,kf.
13	"Epidemiologic Studies"/ or ((epidemiologic* adj (studies or study)) or epidemiology).tw,kf.
14	4 or 5 or 6 or 7 or 8 or 9 or 10 or 11 or 12 or 13
15	3 and 14
16	(exp "Animals"/ or exp "Plants"/) not "Humans"/
17	15 not 16
