# Supplementary material for: Effectiveness and safety of treatments for new-onset, refractory and relapsed vitreoretinal lymphoma: protocol for a systematic review and network meta-analysis
Source: BMJ Open. 2026 Oct 8;16(10):e115420. doi: 10.1136/bmjopen-2025-115420 (PMC13656148; doi:10.1136/bmjopen-2025-115420)
Supplement: Supplementary file 2 [file bmjopen-2025-115420.reviewer_comments.pdf]

PEER REVIEW HISTORY

BMJ Open publishes all reviews undertaken for accepted manuscripts. Reviewers are asked to complete a checklist review form and are provided with free text boxes to elaborate on their assessment. These free text comments are reproduced below.

ARTICLE DETAILS

Title (Provisional)

Effectiveness and safety of treatments for new onset, refractory, and relapsed vitreoretinal lymphoma: protocol for a systematic review and network meta-analysis

Authors

Shields, Melissa; Woodman, Richard; Lake, Stewart; Downie, Laura Elizabeth; Smith, Justine R.

VERSION 1 - REVIEW

**Reviewer**	**1**
**Name**	**Quiroz-Reyes, Miguel A.**
**Affiliation**	**National Autonomous University of Mexico**
**Date**	**22-Mar-2026**
**COI**	**None**

Comments to the authors

RE: Effectiveness and safety of treatments for new onset, refractory, and relapsed vitreoretinal lymphoma: a systematic review and network meta-analysis protocol

Dear Authors, Thank you for letting me read and share my thoughts on this interesting systematic review and network meta-analysis protocol. It carefully looks at how well and safely current treatments work for people who have just been diagnosed with, have refractory, or have recurrent vitreoretinal lymphoma. I like how clearly you laid out your goals, methods, levels of evidence, risk of bias, and the full statistical analyses, which included subgroup and sensitivity analyses. Because there isn't much research on this topic, your work, the first network meta-analysis that compares a range of treatments, such as immunotherapies and systemic therapies, adds important new information and practical advice for treating this uncommon and difficult eye disease. I have ideas that could make your study more scientifically sound and have a bigger effect on the field of ophthalmology.

Introduction.

1. Please think about adding some numbers about survival and making them stand out for their clinical importance.

2. It would also be helpful to explain how diagnostic delays and suboptimal therapy are related by putting together specific outcomes like vision loss, progression to the central nervous system, and death into a single explanation instead of listing them separately.

3. If you can, include numbers about how often or how common it is.

4. Specifically, explain in more detail what is unclear and contradictory about the evidence we have now.

5. If possible, separate the questions about eye control from those about neurological control. Make it clear that there are various studies and reviews with differing conclusions regarding the benefits of systemic therapy for neurological prophylaxis. These differences show that different populations, treatments, and study designs are not the same.

6. In your introduction, talk about the new and targeted therapies and put them in a better light.

Methods.

7. To help readers, clearly put together a summarized PICOS table, since its criteria are all over the place.

8. Please make it clear if the definitions used are the authors' or if you changed them to fit your own definitions. Please ensure that the method definitions are used consistently throughout the text by standardizing them.

9. It is important to clarify the categories of neurological involvement, such as whether isolated vitreoretinal lymphoma (without CNS involvement) at baseline was included.

10. In the intervention section, explain how you will manage combined ocular and systemic therapy to make it clear whether they will be separate nodes on your NMA.

11. Furthermore, make the treatment networks better so that dose intensity or schedule changes are clearer.

12. Please explain why you think it's a good idea to group drugs by how they work and how similar their effects are.

13. In the comparators section, please make it clear if "no treatment" and "best supportive care" will be grouped or kept separate.

14. Please think about making your definitions of outcomes the same.

15. Please make your pre-defined primary and secondary outcomes clearer and more detailed.

16. Please make it clear if you will get hazard ratios for time-to-event results.

17. For each main outcome, please clearly state the minimum important differences, such as the percentage increase in CR, the number of months of PFS/OS, and the absolute increase in AE risk.

18. In the case of adverse events, please make it clear if you will look at ocular-specific AEs separately from systemic AEs.

19. Please make it clear, in light of a few publications, whether you would include gray literature in this review.

20. Please make it clear that you will use GRADE for each important outcome.

21. Could you please explain whether treatments for first-line, refractory, and relapsed patients will be put into separate networks or combined into one network with nodes for each line of therapy? This will help us make sure we do the method right.

22. Combining data and doing statistical analysis.

23. Please make it clear if the analyses were done on a per-patient or per-eye basis.

24. The type of effect is used to measure the main effect in the analysis. In the NMA model, explain how you will deal with correlations when one study gives you more than one comparison result.

25. Please include the details of the NMA software in the appropriate section. Would you?

26. There should be a better explanation of the problems with heterogeneity and how they will affect the results and their meaning.

27. Were there any planned explicit subgroup analyses based on disease status, disease extent, type of local therapy, or novel versus conventional systemic chemotherapy?

28. Include information about what you left out in your sensitivity analyses.

29. You should set up your treatment study nodes ahead of time.

30. You can make your study more scientifically sound by connecting your GRADE to your statistical analysis.

31. I found the following specific limitations that need to be addressed appropriately in the corresponding section: rarity of the studied condition and small sample sizes limiting the statistical power, estimates, and strength of the subgroups; predominance of non-RCTs and retrospective studies subject to confounding, selection bias, and information bias leading in consequence to a low or very low certainty of evidence in most of your comparisons; high heterogeneity and the presence of strong effect modifiers that might violate the assumptions of your well-conducted NMA; your manuscript has plenty of variable diagnostic criteria and outcome definitions; please address them to increase the scientific value; there is heterogeneity on treatments and dosing grouped in single nodes that might obscure differences and contribute even more to heterogeneity between studies. There isn't much information on new therapies, which could make it hard to make strong comparisons. This could cause the NMA to rank these treatments as reliable, but there is a higher chance of publication and reporting bias. Please talk about this important issue. Another problem is that it can't be used in settings with different resources, supportive care, or patient

demographics. Furthermore, it's hard to fully understand how time and practice have changed because older groups may not be directly comparable to newer studies that use modern imaging, molecular diagnostics, and targeted therapies.

Conclusion.

32. Your conclusion section is well-organized, but you could make it even stronger by adding a clear gap or background on the problem you are looking into.

33. In your conclusion, say what this review has added to what we already know about the condition you studied, and make it clear how effective and safe it is.

34. One sentence should be added about the expected impact and how the finding will be used, with an emphasis on developing guidelines, making clinical decisions, and finding research gaps.

VERSION 1 - AUTHOR RESPONSE

**REVIEWER 1**	
**Dear Authors, Thank you for letting me read and share my thoughts on this interesting systematic review and network meta-analysis protocol. It carefully looks at how well and safely current treatments work for people who have just been diagnosed with, have refractory, or have recurrent vitreoretinal lymphoma. I like how clearly you laid out your goals, methods, levels of evidence, risk of bias, and the full statistical analyses, which included subgroup and sensitivity analyses. Because there isn't much research on this topic, your work, the first network meta-analysis that compares a range of treatments, such as immunotherapies and systemic therapies, adds important new information and practical advice for treating this uncommon and difficult eye disease. I have ideas that could make your study more scientifically sound and have a bigger effect on the field of ophthalmology.**	Response: We appreciate the thoughtful feedback. We have carefully addressed your concerns, and we believe the manuscript has been significantly strengthened as a result.
**Introduction.**<br><br>**1. Please think about adding some**	Response: We appreciate this recommendation. The poor survival statistics highlight the limitations of current treatment. The following

**numbers about survival and making them stand out for their clinical importance.**	sentences have been added to our introductory paragraph (Page 3, Lines 62-68):<br><br>"Vitreoretinal lymphoma exhibits a variable pattern of central nervous system (CNS) involvement that may precede, coincide with, or follow the ocular disease, and ultimately occurs in an estimated 69% of patients.(4) Conversely, VRL occurs in about one in six patients with PCNSL.(4) When VRL is confined to the eye at diagnosis, termed primary VRL (PVRL), around 50% of individuals experience progression of the cancer into the CNS.(5) Overall survival remains poor, with the 5-year survival measured at approximately 40% in population-based studies.(6,7)"
**2. It would also be helpful to explain how diagnostic delays and suboptimal therapy are related by putting together specific outcomes like vision loss, progression to the central nervous system, and death into a single explanation instead of listing them separately.**	Response: The below sentence has been added to the Introduction to discuss how delaying the diagnosis of VRL leads to suboptimal outcomes (Page 3, Lines 73-77).<br><br>"Vitreoretinal lymphoma frequently masquerades as inflammatory eye disease, leading to diagnostic delays and misdiagnosis that further contribute to a poor prognosis.(1,2,5) Treatment delays are associated with higher disease burden, including retinal and subretinal infiltration, that are significant predictors of vision loss(10,11) and may be associated with shorter mean survival time.(12)"
**3. If you can, include numbers about how often or how common it is.**	Response: We have the following sentences outlining disease rarity, global prevalence rate and the rising incidence over recent decades (Page 3, Lines 70-73).<br><br>"Although VRL is the most common intraocular lymphoma, it is a rare

	cancer, accounting for less than 3% of all ocular tumours.(8) The 10-year cumulative prevalence of PVRL was estimated at 0.076 per 100,000 persons using data from the TriNetX clinical database.(9)”
**4. Specifically, explain in more detail what is unclear and contradictory about the evidence we have now.**	Response: We appreciate the suggestion as it is central to justifying our systematic review. In the Introduction we now discuss the contradictory evidence around the use of systemic chemotherapy to prevent or delay CNS progression (Page 4, Lines 109-116). A paragraph on the limitations of the current evidence has also been added to the introduction (Page5, Lines 138-143).
**5. If possible, separate the questions about eye control from those about neurological control. Make it clear that there are various studies and reviews with differing conclusions regarding the benefits of systemic therapy for neurological prophylaxis. These differences show that different populations, treatments, and study designs are not the same.**	Response: The manuscript now presents the eye and neurological control issues in separate paragraphs, beginning with local control and concerns about ocular and CNS relapse. We note that initial control may be achieved with either local chemotherapy or radiotherapy, and discuss common adverse events for each option (Page 4, Lines 90-105). We also elaborate on the conflicting evidence surrounding systemic chemotherapy for CNS prophylaxis (Page 4, Lines 109-116).
**6. In your introduction, talk about the new and targeted therapies and put them in a better light.**	Response: Thank you for the recommendation. Examining how new and targeted therapies compare with more established treatments is a primary rationale for undertaking our systematic review and NMA. The introduction has been updated to include a paragraph detailing available new and targeted therapies for VRL (Page 5, Lines 123-136).
**Methods.**	Response: Thank you for the suggestion. Our methodology is now

**7. To help readers, clearly put together a summarized PICOS table, since its criteria are all over the place.**	outlined in a PICO table (Table 1) (Page 21, Line 561).
**8. Please make it clear if the definitions used are the authors' or if you changed them to fit your own definitions. Please ensure that the method definitions are used consistently throughout the text by standardizing them.**	<u>Response:</u> Outcome definitions are not our own; they are taken from published, peer-reviewed literature in VRL research. Care has been taken to standardise outcome definitions across our manuscript and reference appropriately (Page 9, Line 229-256).
**9. It is important to clarify the categories of neurological involvement, such as whether isolated vitreoretinal lymphoma (without CNS involvement) at baseline was included.**	<u>Response:</u> We will categorise neurological involvement into two groups: VRL with no CNS involvement at baseline (also termed primary VRL) and VRL with neurological involvement at baseline. A planned subgroup analysis between these groups is now clearly described (Page 17, Line 466-467).
**10. In the intervention section, explain how you will manage combined ocular and systemic therapy to make it clear whether they will be separate nodes on your NMA.**	<u>Response:</u> Treatment categories/nodes are defined in our manuscript to show how ocular and systemic therapy will be handled (Page 15, Line 405-420). Combined therapies have their own pre-specified categories (i.e. high-dose intravenous methotrexate and intravitreous methotrexate). In cases of sparse data, local therapies (ocular radiotherapy and/or ocular chemotherapy) will be aggregated into the category “Local eye-directed therapy”; all systemic chemotherapy will be aggregated into “Systemic Chemotherapy”; and combinations of ocular and systemic treatment will be grouped as “Combined” composite nodes. This has been specified in the manuscript (Page 15, Line 415-420).
**11. Furthermore, make the treatment networks better so that dose intensity or schedule changes are clearer.**	<u>Response:</u> Dosing and administration frequency are often undefined in VRL treatment studies. We will accept any regime for a particular treatment type that includes at least one dose of that treatment. This has been clarified in

	the manuscript (Page 15, Lines 411-412).
**12. Please explain why you think it's a good idea to group drugs by how they work and how similar their effects are.**	Response: There is precedent in the literature on VRL for combining drugs by class as well as by local versus systemic action. Given small study sizes, most studies tend to combine like treatment arms. In prior research (Riemans et al., 2015; Raval et al., 2021), studies often include a local arm (intravitreal methotrexate, intravitreal rituximab, and ocular radiotherapy), a systemic arm, and a combined ocular plus systemic arm. If sample sizes permit, individual BTK inhibitors (ibrutinib and zanubrutinib) will be treated as separate nodes. We will pool across BTK inhibitors and perform a class-level analysis only when numbers are insufficient for individual analyses (Page 15, Lines 413-415).
**13. In the comparators section, please make it clear if "no treatment" and "best supportive care" will be grouped or kept separate.**	Response: These groups will be combined and the following sentence has been added to the methods for clarity (Page 8, Lines 205-207):<br><br>“No-treatment and best supportive care will be grouped together as a single comparator for the analysis.”
**14. Please think about making your definitions of outcomes the same.**	Response: Our outcomes and definitions have been standardised across the manuscript. Please refer to Reviewer query 8 (above) for discussion.
**15. Please make your pre-defined primary and secondary outcomes clearer and more detailed.**	Response: The outcomes section has been restructured to improve presentation of primary and secondary outcomes. Definitions for each outcome have been standardised and appropriately referenced (Page 9, Lines 229-256).

**16. Please make it clear if you will get hazard ratios for time-to-event results.**	Response: The following sentence has been added for clarity (Page 15, Lines 401-402):<br><br>"Time-to-event outcomes will use pooled HRs when possible."
**17. For each main outcome, please clearly state the minimum important differences, such as the percentage increase in CR, the number of months of PFS/OS, and the absolute increase in AE risk.**	Response: To date, there are no established MCID thresholds for defining survival benefit in VRL. Frameworks such as ESMO-MCBS and ASCO-VF have been used to set thresholds in other cancers with large study volumes (e.g., lung cancer, Fu et al, >300 RCTs), but these thresholds have not been defined for VRL, highlighting a gap and a direction for future research. In VRL, MCID has been applied to patient-reported outcomes including quality of life, symptoms, and Best Corrected Visual Acuity (5–7 letters), with HRQoL thresholds (5–10 points on EORTC QLQ-C30) cited in the literature on PCNSL (Luangmaneecrotea et al). Neither set of outcomes is the primary focus of our systematic review. Therefore, results will be reported based on statistically significant improvements in OS and PFS, rather than pre-specified MCID thresholds.<br><br>Please find the full citation below for the references mentioned above.<br><br>Fu YL, Tang ZY, Zhu YY, Liu QW, Zhang MY, Gu C, Deng Y, Li S, Zhang JX, Li JB. Clinical significance and minimal clinically important differences for the survival outcomes in randomised clinical trials of lung cancer. BMC Cancer. 2025 Nov 5;25(1):1712. doi: 10.1186/s12885-025-15169-7. PMID: 41194034; PMCID: PMC12587515.<br><br>Luangmaneerote W, Rattanapong N, Chokesuwattanaskul A, Raiyawa T,

	Chajuwan T, Jongdee P, Rojnuckarin P, Chanswangphuwana C. Real-world survival, cognitive, and quality of life outcomes of methotrexate/ifosfamide plus whole brain radiotherapy for newly diagnosed primary central nervous system lymphoma. Ther Adv Hematol. 2025 Dec 14;16:20406207251400429. doi: 10.1177/20406207251400429. PMID: 41404495; PMCID: PMC12703023.
**18. In the case of adverse events, please make it clear if you will look at ocular-specific AEs separately from systemic AEs.**	Response: Ocular specific and systemic adverse events will be considered separately (Page 9, Line 246).
**19. Please make it clear, in light of a few publications, whether you would include gray literature in this review.**	Response: The search strategy will focus on peer-reviewed literature. Grey literature will not be systematically sought. The following sentence has been added to the manuscript: “Grey literature will not be sourced for our review” (Page 10, Line 285-286).
**20. Please make it clear that you will use GRADE for each important .**	Response: For each outcome, the certainty of evidence will be assessed using the GRADE framework. This paragraph has been updated in the manuscript (Pages 13 and 14, Lines 366-374).
**21. Could you please explain whether treatments for first-line, refractory, and relapsed patients will be put into separate networks or combined into one network with nodes for each line of therapy? This will help us make sure we do the method right.**	Response: Treatments for first-line, relapsed and refractory patients will be placed into separate networks. The following sentence has been added to the manuscript (Page 16, Lines 429-430):<br><br>“Treatments for new onset, relapsed and refractory cases will be placed into separate networks.”
**22. Combining data and doing statistical analysis.**	Response: We interpret this point as a heading introducing the subsequent feedback points.

**23. Please make it clear if the analyses were done on a per-patient or per-eye basis.**	<u>Response:</u> Analyses will be done on a per-patient basis. The following sentence has been added to the manuscript (Page 14, Lines 394-395):<br><br>"Analysis will be done on a per-patient basis."
**24. The type of effect is used to measure the main effect in the analysis. In the NMA model, explain how you will deal with correlations when one study gives you more than one comparison result.**	<u>Response:</u> When a study reports more than one eligible comparison, we will choose the primary effect in accordance with our prespecified outcome measures. The following sentence has been added to our manuscript (Page 14, Lines 391-393):<br><br>"When a study reports more than one eligible comparison, the primary effect will be designated in accordance with the prespecified primary outcomes to avoid within-study dependence."
**25. Please include the details of the NMA software in the appropriate section. Would you?**	<u>Response:</u> The meta-analyses will be conducted in Stata (19.1, StataCorp LLC, College Station, TX). This has been incorporated into the manuscript (Page 14, Lines 395-396).
**26. There should be a better explanation of the problems with heterogeneity and how they will affect the results and their meaning.**	<u>Response:</u> We thank the reviewer for highlighting this important point, as heterogeneity is a major issue affecting research on VRL. In treatment studies, where data are often small and heterogeneous, we will use a random-effects model to account for between-study variability. We will quantify heterogeneity using I^2 and τ^2 and interpret these alongside the pooled effect estimates. To identify sources of heterogeneity, we will perform meta-regression analyses with moderators such as study design (retrospective vs prospective), follow-up duration, and risk-of-bias indicators. We have added two new paragraphs to our methods under the heading "Heterogeneity and

	Transitivity Assessment" to address these concerns (Pages 16 and 17, Lines 444-459).
**27. Were there any planned explicit subgroup analyses based on disease status, disease extent, type of local therapy, or novel versus conventional systemic chemotherapy?**	<u>Response:</u> Thank you for these suggestions. We have specified our planned subgroup analyses based on your recommendations in a new paragraph entitled "Subgroup Analysis" (Page 17, Lines 461-472).
**28. Include information about what you left out in your sensitivity analyses.**	<u>Response:</u> The following sentence has been added to our manuscript (Page 17, Lines 477-479).<br><br>"A list of studies that have been excluded will be provided in the supplementary materials that accompany the publication of the systematic review and NMA."
**29. You should set up your treatment study nodes ahead of time.**	<u>Response:</u> Establishing treatment nodes a priori is challenging because the number of studies contributing data to each arm is not known in advance. A treatment option will be represented as a node only if there are at least two studies evaluating the treatment at a comparable dose and regimen.<br><br>At a minimum, we anticipate nodes for the following treatments, with sufficient data expected: intravitreal methotrexate monotherapy; intravitreal rituximab monotherapy; ocular radiation monotherapy; high-dose intravenous methotrexate-based chemotherapy monotherapy; intravitreal methotrexate in combination with high-dose intravenous methotrexate; ocular radiotherapy with high-dose intravenous methotrexate-based chemotherapy; Bruton's tyrosine kinase inhibitors. Additional nodes may be added as data becomes

	available. This clarification is noted in the manuscript (Page 15, Lines 405-420).
**30. You can make your study more scientifically sound by connecting your GRADE to your statistical analysis.**	Response: The below statement has been added to the 'Data synthesis and statistical analysis' section (Page 14, Lines 393-394):<br><br>"The primary analysis will include all studies regardless of GRADE score. A secondary analysis will exclude studies judged to have a high risk of bias."
**31. I found the following specific limitations that need to be addressed appropriately in the corresponding section: rarity of the studied condition and small sample sizes limiting the statistical power, estimates, and strength of the subgroups; predominance of non-RCTs and retrospective studies subject to confounding, selection bias, and information bias leading in consequence to a low or very low certainty of evidence in most of your comparisons; high heterogeneity and the presence of strong effect modifiers that might violate the assumptions of your well-conducted NMA; your manuscript has plenty of variable diagnostic criteria and outcome definitions; please address them to increase the scientific value; there is heterogeneity on treatments and dosing grouped in single nodes that might obscure differences and contribute even more to heterogeneity between studies. There isn't much information on new therapies, which could make it hard to make strong comparisons. This could cause the NMA to rank these treatments as reliable, but there is a higher chance of publication and reporting bias. Please talk about this important issue. Another problem is that it can't be used in settings with different resources, supportive care,**	Reviewer: *"I found the following specific limitations that need to be addressed appropriately in the corresponding section: rarity of the studied condition and small sample sizes limiting the statistical power, estimates, and strength of the subgroups; predominance of non-RCTs and retrospective studies subject to confounding, selection bias, and information bias leading in consequence to a low or very low certainty of evidence in most of your comparisons; high heterogeneity and the presence of strong effect modifiers that might violate the assumptions of your well-conducted NMA;"*<br><br>Response: We thank the reviewer for making these excellent points. Rarity, small sample sizes, non-RCT predominance and heterogeneity are significant issues affecting research into VRL treatments. To address these issues as rigorously as possible, we plan to implement the following strategies; (1) A random-effects framework with heterogeneity quantification (I^2, τ^2) (Page 16, Lines 444-449) plus prespecified meta-regression and subgroup analyses (Page 17, Lines 461 – 472); (2)

**or patient demographics. Furthermore, it's hard to fully understand how time and practice have changed because older groups may not be directly comparable to newer studies that use modern imaging, molecular diagnostics, and targeted therapies.**	Comprehensive and exhaustive search of the literature, including thorough reference mining of existing papers (Page 10, Lines 277-279); (3) Careful review of outcome definitions and treatment regimens across included papers to ensure cross-study comparability. When feasible, move beyond labelling a single "primary treatment" to enable equitable comparisons of regimens. We have selected primary outcomes that are relatively standardised across studies (e.g., OS and PFS) (Page 9, Lines 230-235); (4) Predefining treatment categories to distinguish local vs. systemic therapies and accommodate common combinations (e.g., intravitreal plus systemic methotrexate), reducing cohort heterogeneity (Page 15, Line 405-411). (6) Risk-of-bias assessments (Page 13, Line 353-364); (7) If data permit, conducting sensitivity analyses to evaluate the impact of excluding studies with a high risk of bias (Page 17, Lines 474-479); (8) Pre-registration of our protocol to ensure full, transparent reporting of outcomes (Page 6, Line 163-164).<br><br><u>Reviewer:</u> *"your manuscript has plenty of variable diagnostic criteria and outcome definitions; please address them to increase the scientific value"*<br><br><u>Response:</u> We have standardised the diagnostic criteria and outcome definitions across our manuscript to enhance consistency and comparability. Our outcome definitions can be found at (Page 9, Lines 229-256).

	<u>Reviewer:</u> *“There isn't much information on new therapies, which could make it hard to make strong comparisons. This could cause the NMA to rank these treatments as reliable, but there is a higher chance of publication and reporting bias.”*<br><br><u>Response:</u> We thank the reviewer for highlighting this and agree that sparse data networks for novel therapies could lead to overoptimistic ranking results. To address this in our final analysis, we have implemented the following safeguards in our protocol: (1) Quality of Evidence Assessments: We will use the CINeMA (Confidence in Network Meta-Analysis) framework or GRADE-NMA to explicitly rate the certainty of evidence for each pairwise comparison. This tool directly penalises "reporting bias" and "imprecision," ensuring that highly ranked treatments with little data are flagged as having low/very low certainty (Pages 13-14, Lines 366-374); (2) Bias Evaluation: We will utilise network funnels (comparison-adjusted funnel plots) to visually inspect the network for small-study effects and publication bias. We have updated the “Assessment of Risk of Bias in Included Studies and Certainty of Body of Evidence” section (Page 14, Line 376-380) of the protocol to detail these specific steps, and we have expanded our discussion to explicitly note the risk of small nodes for novel therapies as a potential limitation (Page 19, Lines 526-535).<br><u>Reviewer:</u> *"Another problem is that it can't be used in settings with different resources, supportive care, or patient demographics."*<br><br><u>Response:</u> The reviewer makes an excellent point regarding the

	generalisability and the underlying transitivity assumption of our NMA. Variations in background supportive care, healthcare infrastructure, and patient demographics are effect modifiers in VRL. We have addressed this limitation and clarified our handling of these variables as follows: (1) Strict eligibility criteria: To preserve transitivity, our protocol enforces rigorous inclusion criteria regarding trial design and baseline patient characteristics (Pages 6-7, Line 170-179). (2) Network meta-regression and subgroup analyses: Where data permit, we will perform network meta-regression or stratified subgroup analyses based on key effect modifiers, specifically looking at geographic region (as a proxy for resource settings/supportive care standards) and baseline demographic variables (e.g., median age, line of therapy) (Page 17, Lines 461-472).<br>We have added a dedicated paragraph outlining how we will evaluate heterogeneity across settings ("Heterogeneity and Transitivity Assessment" (Page 16, Lines 444-459)) and have noted this as a key consideration in our discussion (Page 19, Lines 512-524).<br><br><br>Reviewer: *"Furthermore, it's hard to fully understand how time and practice have changed because older groups may not be directly comparable to newer studies that use modern imaging, molecular diagnostics, and targeted therapies."*<br><br>Response: We thank the reviewer for this important point. We acknowledge that temporal changes in diagnostics and management can affect cross-study comparability in VRL research.

	To mitigate this, we restrict our literature search to studies published from 2000 onward (aligned with ICD code changes enacted in 2000) and this era boundary is justified in our methods (Page 10, Line 277-280). We also added a dedicated discussion of era effects in the limitations of research in this field, including older cohorts potentially being differently classified or less representative due to evolving ocular imaging and molecular diagnostics such as tumor gene testing and cytokine analysis (Page 18, Lines 503-511).
**Conclusion.**<br><br>**32. Your conclusion section is well-organized, but you could make it even stronger by adding a clear gap or background on the problem you are looking into.**	<u>Response:</u> Thank you for the suggestion to articulate a clearer gap in the literature to strengthen the framing of our manuscript. In line with BMJ Open editorial guidance that protocols do not include formal conclusions, we have addressed this feedback in the Introduction (Beginning Page 3, Line 57) and new Discussion section (Beginning Page 18, Line 496).
**33. In your conclusion, say what this review has added to what we already know about the condition you studied, and make it clear how effective and safe it is.**	<u>Response:</u> These are important points to address. We have rewritten our Introduction to emphasise them (Beginning Page 3, Line 57).
**34. One sentence should be added about the expected impact and how the finding will be used, with an emphasis on developing guidelines, making clinical decisions, and finding research gaps.**	<u>Response:</u> Thank you for this suggestion. The following sentence has been added to the Discussion (Page 19, Lines 537-541):<br><br>“This systematic review and NMA will evaluate the effectiveness and safety of treatments for new onset, relapsed, and refractory VRL. The NMA should, in particular, provide an effectiveness and safety hierarchy that can serve as a decision-support tool, and perhaps boost interest in the development of

	clinical guidelines that can be used across global resource settings."

VERSION 2 - REVIEW

**Reviewer**	**1**
**Name**	**Quiroz-Reyes, Miguel A.**
**Affiliation**	**National Autonomous University of Mexico**
**Date**	**22-Jul-2026**
**COI**	

I have reviewed your manuscript and observed that some of my comments and suggestions remain only partially addressed. The following recommendations should be addressed in more detail:

4. Clarify what is unclear/contradictory about current evidence—partially addressed.

You show conflicting evidence on systemic therapy (systemic vs ocular only, differing CNS progression vs OS) and scattered regimens, but this is mostly in the later part of the introduction/discussion. If you want to be very explicit, you could add one synthesis sentence early in the introduction clearly stating that existing data are conflicting and methodologically heterogeneous.

8. Clarify whether definitions are author-derived or adapted; standardize them—Partially addressed.

You define VRL, PVRL, relapsed, refractory, and CNS relapse quite clearly. However, you do not always say whether you will adopt authors' definitions versus reclassifying based on your own. You do say you will harmonize outcome definitions based on narrative descriptions, which goes in this direction. If you want to be fully responsive, you might add a sentence in the Outcomes/Methods stating how you will handle and standardize heterogeneous author definitions (e.g., relapse, CR/PR, CNS progression).

11. Improve treatment networks regarding dose intensity/schedule – Partially addressed.

You state that treatment categories will be used only when at least two studies report comparable doses/regimens and will be grouped by common regimens. That acknowledges the dose/regimen but doesn't fully spell out how you'll handle different

intensities/schedules within a category. Consider one line saying you will, where necessary, stratify or conduct sensitivity analyses when materially different dose intensity/schedules exist.

17. Minimum important differences for each main outcome – Not clearly addressed.

I don't see specific thresholds (e.g., % increase in CR, months of PFS/OS, AE risk increments) defined as minimally important differences. You describe time points and effect measures, but not what change is considered clinically important. If the journal expects this to be specified, you should add a short paragraph (even if partly based on expert consensus) in the Outcomes/Statistical Analysis section.

18. Clarify whether ocular-specific AEs will be separate from systemic AEs—Partially addressed.

You say both ocular and systemic events will be considered, but you don't clearly say you'll analyze them separately. A short sentence stating that ocular and systemic AEs will be reported/analyzed separately would fully respond.

VERSION 2 - AUTHOR RESPONSE

4. Clarify what is unclear/contradictory about current evidence – **Partially addressed.**

You show conflicting evidence on systemic therapy (systemic vs ocular-only, differing CNS progression vs OS) and scattered regimens, but this is mostly in the later part of the Introduction/Discussion. If you want to be very explicit, you could add one synthesis sentence early in the Introduction clearly stating that existing data are conflicting and methodologically heterogeneous.

8. Clarify whether definitions are author-derived or adapted; standardize them – **Partially addressed.**

You define VRL, PVRL, relapsed, refractory, and CNS relapse quite clearly. However, you do not always say whether you will *adopt* authors' definitions versus *reclassify* based on your own. You do say you will harmonize outcome definitions based on narrative descriptions, which goes in this direction. If you want to be fully responsive, you might add a sentence in the Outcomes/Methods stating how you will handle and standardize heterogeneous author definitions (e.g., relapse, CR/PR, CNS progression).

11. Improve treatment networks regarding dose-intensity/schedule – **Partially addressed.**

You state that treatment categories will be used only when at least two studies report comparable doses/regimens and will be grouped by common regimens. That acknowledges the dose/regimen but doesn't fully spell out how you'll handle different intensities/schedules

within a category. Consider one line saying you will, where necessary, stratify or conduct sensitivity analyses when materially different dose-intensity/schedules exist.

17. Minimum important differences for each main outcome – **Not clearly addressed.**

I don't see specific thresholds (e.g., % increase in CR, months of PFS/OS, AE risk increments) defined as minimally important differences. You describe time points and effect measures, but not what change is considered clinically important. If the journal expects this to be specified, you should add a short paragraph (even if partly based on expert consensus) in the Outcomes/Statistical Analysis section.

18. Clarify whether ocular-specific AEs will be separate from systemic AEs – **Partially addressed.**

You say both ocular and systemic events will be considered, but you don't clearly say you'll *analyze them separately*. A short sentence stating that ocular and systemic AEs will be reported/analysed separately would fully respond.

VERSION 3 - REVIEW

**Reviewer**	**1**
**Name**	**Quiroz-Reyes, Miguel A.**
**Affiliation**	**National Autonomous University of Mexico**
**Date**	**01-Sep-2026**
**COI**	

Final comments on the latest revision of the paper

Title. Effectiveness and safety of treatments for new onset, refractory, and relapsed vitreoretinal lymphoma: protocol for a systematic review and network meta-analysis

Dear Editor and Authors,

Most of my remaining issues have been addressed in the latest amended text, but comment 17 remains the weakest response.

You should bolster the reply to my comment 17. I'd recommend making the paragraph clearer about how clinical importance would be considered, as the existing wording is forceful yet relatively generic. In particular, it could be enhanced by adding a tighter version like:

Since there are no established VRL-specific minimally important difference thresholds, we will not employ set disease-specific thresholds to determine clinical relevance. Instead, we will interpret the treatment effects in a pragmatic, expert-informed framework that incorporates the magnitude and precision of effect estimates, baseline risk, certainty of the

evidence, and the predicted balance of benefit and harm. Effects suggesting a meaningful improvement in overall survival or progression-free survival will be considered to have greater clinical importance, especially when little or no effect can be excluded by confidence intervals, and the results are consistent across study designs or disease subgroups. For ocular response outcomes, larger complete or partial response rates would be considered clinically relevant if responses are sustained and associated with reduced relapse, reduced CNS progression, or maintenance of visual function where documented. For safety outcomes, increases in CTCAE Grade 3–4 adverse events will be deemed clinically relevant if they represent a material increase in treatment burden, hospitalization, treatment stoppage, or risk of life-threatening toxicity. Where data allow, these judgments will be applied individually for newly diagnosed, refractory and relapsed VRL groups.

VERSION 3 - AUTHOR RESPONSE

REVIEWER 1	Author response
**Most of my remaining issues have been addressed in the latest amended text, but comment 17 remains the weakest response.**<br><br>**You should bolster the reply to my comment 17. I'd recommend making the paragraph clearer about how clinical importance would be considered, as the existing wording is forceful yet relatively generic. In particular, it could be enhanced by adding a tighter version like:**<br><br>**Since there are no established VRL-specific minimally important difference thresholds, we will not employ set disease-specific thresholds to determine clinical relevance. Instead, we will interpret the treatment effects in a pragmatic, expert-informed framework that incorporates the magnitude and precision of effect estimates, baseline risk, certainty of the evidence, and the predicted balance of benefit and harm. Effects suggesting a meaningful improvement in overall survival or progression-free survival will be considered to have greater clinical importance, especially when little or no effect can be excluded by confidence**	Response: The paragraph on minimally important differences has been expanded as per the reviewer's recommendation. (Pages 10-11, Lines 285 - 299)<br><br>In preparing this revision, we realized that ocular-specific adverse event definitions had not been included; we have now added these to the manuscript. (Page 9, Lines 254 - 260)<br><br>The CTCAE reference (Reference 45) has been updated to reflect the updated 2025 version. (Page 26, Line 751)

**intervals, and the results are consistent across study designs or disease subgroups. For ocular response outcomes, larger complete or partial response rates would be considered clinically relevant if responses are sustained and associated with reduced relapse, reduced CNS progression, or maintenance of visual function where documented. For safety outcomes, increases in CTCAE Grade 3–4 adverse events will be deemed clinically relevant if they represent a material increase in treatment burden, hospitalization, treatment stoppage, or risk of life-threatening toxicity. Where data allow, these judgments will be applied individually for newly diagnosed, refractory and relapsed VRL groups.**<br><br>**COMMENT 17**<br>**“For each main outcome, please clearly state the minimum important differences, such as the percentage increase in CR, the number of months of PFS/OS, and the absolute increase in AE risk.”**	
